# When knowledge hurts: humans are willing to receive pain for obtaining non-instrumental information

**DOI:** 10.1098/rspb.2023.1175

**Published:** 2023-07-12

**Authors:** Stefan Bode, Xiaoyu Sun, Matthew Jiwa, Patrick S. Cooper, Trevor T.-J. Chong, Natalia Egorova-Brumley

**Affiliations:** ^1^ Melbourne School of Psychological Sciences, The University of Melbourne, Melbourne 3010, Australia; ^2^ Turner Institute for Brain and Mental Health, Monash University, Clayton 3080, Australia; ^3^ Department of Neurology, Alfred Health, Melbourne 3004, Australia; ^4^ Department of Clinical Neurosciences, St Vincent's Hospital, Melbourne 3065, Australia

**Keywords:** information-seeking, non-instrumental information, pain, information value, uncertainty

## Abstract

Humans and other animals value information that reduces uncertainty or leads to pleasurable anticipation, even if it cannot be used to gain tangible rewards or change outcomes. In exchange, they are willing to incur significant costs, sacrifice rewards or invest effort. We investigated whether human participants were also willing to endure pain—a highly salient and aversive cost—to obtain such information. Forty participants performed a computer-based task. On each trial, they observed a coin flip, with each side associated with different monetary rewards of varying magnitude. Participants could choose to endure a painful stimulus (low, moderate or high pain) to learn the outcome of the coin flip immediately. Importantly, regardless of their choice, winnings were always earned, rendering this information non-instrumental. Results showed that agents were willing to endure pain in exchange for information, with a lower likelihood of doing so as pain levels increased. Both higher average rewards and a larger variance between the two possible rewards independently increased the willingness to accept pain. Our results show that the intrinsic value of escaping uncertainty through non-instrumental information is sufficient to offset pain experiences, suggesting a shared mechanism through which these can be directly compared.

## 1. Background

The drive to understand the world and actively seek information is an important aspect of human (and other animal) cognition [1–3]. Knowledge about what will happen in the immediate future is of significant value for most animal species, even if this information cannot be used to alter the course of events or to optimize reward outcomes (i.e. is ‘non-instrumental’). For example, non-human primates have been shown to prefer information about the size of an upcoming water reward [4], and they are even willing to sacrifice a proportion of their expected rewards to learn this information [5]. Similar results have been observed for starlings [6] and pigeons [7]. Recent research has shown that humans have similar preferences for non-instrumental information. For example, humans are willing to invest monetary costs [8–11] or physical effort [12] to immediately learn the outcome of pre-determined lotteries. This behaviour suggests that the subjective value of information is determined by more than just its instrumental utility and might be intrinsically rewarding [13]. It could also be driven by cognitive factors, such as the capacity of that information to refine cognitive models of the world by reducing uncertainty [8,11,14], by low confidence in the available information [15,16], and by emotional factors, such as the desire for positive outcomes or belief states [12,14,17–19], and reduction of anxiety [9,20].

It has further been suggested that agents might neurally process information in a ‘common currency’ to primary rewards [21,22]. In support, midbrain dopamine neurons in monkeys have been shown to signal both expected reward and expected information [4], and encode both reward prediction errors (RPEs; i.e. whether more or less reward was received than anticipated) and information prediction errors (IPEs; i.e. whether more or less information was received than anticipated) [21]. In humans, RPEs and IPEs are encoded in a highly similar fashion in the feedback-related negativity component of the event-related potential (ERP), which is thought to originate from the medial prefrontal areas, including the anterior cingulate cortex (ACC) [10]. In line with this finding, parts of the brain's reward system have been found to overlap to a large degree with information-processing regions, including the ACC, in which both subjective value of information and RPEs are encoded [12,23–25].

But how valuable is non-instrumental information for humans? It could be argued that in previous work, the cost associated with obtaining non-instrumental information was relatively low (e.g. in the range of cents in most lottery tasks [8–11]). While these findings are robust and replicated when physical effort was used, which is an arguably more ecologically valid cost [12], such costs might still not be strongly aversive. In this study, we therefore tested the hypothesis that humans are also willing to accept pain as a cost to obtain non-instrumental information.

Pain is a particularly aversive stimulus with significant biological relevance, as it signals harm, and animals naturally strive to avoid it (e.g. [26–28]). Accordingly, it has been shown that if receiving higher levels of pain is a possible outcome of a gamble, people become more risk averse [29]. Pain has been used as a stimulus for punishment in animal and human experiments for decades. Importantly, it has been suggested that pain is not the result of a simple read-out of a noxious stimulus, but rather a constructive process translating sensory, cognitive and affective aspects into a unified pain experience [30]. Pain perception has been argued to involve a rather complex inference process, and uncertainty in particular enhances pain perception (e.g. [31,32]; for a review see [28]).

It has also been shown that human participants are willing to accept pain in exchange for rewards, and that potential future pain was represented neurally in a network including reward-related brain regions [33]. There is further evidence for a partly overlapping neural system that facilitates the integration of the valuation of pain (and other aversive stimuli) with reward value, which are both modulated by the dopamine and opioid systems [34]. For example, the ventral striatum, which is involved in subjective pain perception, is also a key area of the reward circuit [35], and the orbitofrontal cortex contributes to integrating the value of rewards and punishments for decision-making (e.g. [36]). Dopamine neurons in the midbrain of monkeys that code for rewards also transmit signals related to non-rewarding, aversive experiences, if these have high saliency [37]. In monkeys, the ACC and the strongly interconnected ventrolateral prefrontal cortex (vlPFC), regions that code for valuation of positively valenced information, have been found to also code for negatively valenced information, such as an upcoming aversive stimulus, although in distinct neural populations [26]. This suggests that the ACC and vlPFC might play an important role in integrating preference signals for information in relation to both punishing and rewarding stimuli [26]. Together, this leads to the hypothesis that the negative valence of pain and the positive valence of rewards could be represented within the same system, allowing their intrinsic values to be directly traded off (e.g. [34]). Understanding whether humans accept pain in exchange for information that has no instrumental utility therefore could shed additional light on how the value of information fits into this framework and allows for testing the hypothesis that even non-instrumental information is intrinsically valuable at a fundamental level.

To investigate this question, we tested whether human participants were willing to accept painful stimuli of varying intensities in exchange for immediate information about the outcome of a coin flip lottery. In each trial, participants could choose to receive a painful thermal stimulus to their forearm, calibrated as either subjectively low, moderate or high pain, to learn the mapping between two potential winning amounts and the sides of a coin. These amounts varied in overall expected value (i.e. the average amount that could be won in a given trial) and the range (i.e. the difference between the two sides). Importantly, regardless of whether participants chose to learn the mapping (and hence the amount won), their winnings were always added to their running total, meaning that the information was truly non-instrumental as it did not impact the lottery outcome. We hypothesized that participants would accept at least low levels of pain for non-instrumental information. We further hypothesized that the probability of choosing information would be moderated by both the expected reward magnitude as well as the range between amounts in each lottery, as these factors increase the stakes, and therefore the relevance of the uncertainty and the anticipation of potentially larger winnings, which have both been suggested to drive information-seeking (e.g. [22]).

## 2. Methods

### (a) Participants

Forty healthy human participants (18–37 years; *M* = 23.03, *s.d.* = 4.58; 28 female, 12 male) participated in the study and received AUD15 reimbursement. They were informed that additionally they could win up to AUD10 in the task. Note that we always paid the full AUD25 in the end, which was equivalent to, or slightly above, participants' expectations. Paying this small bonus was not done to deceive participants, who did not know their running total throughout the experiment, but for reasons of equity.

### (b) Stimuli

**Figure 1.**
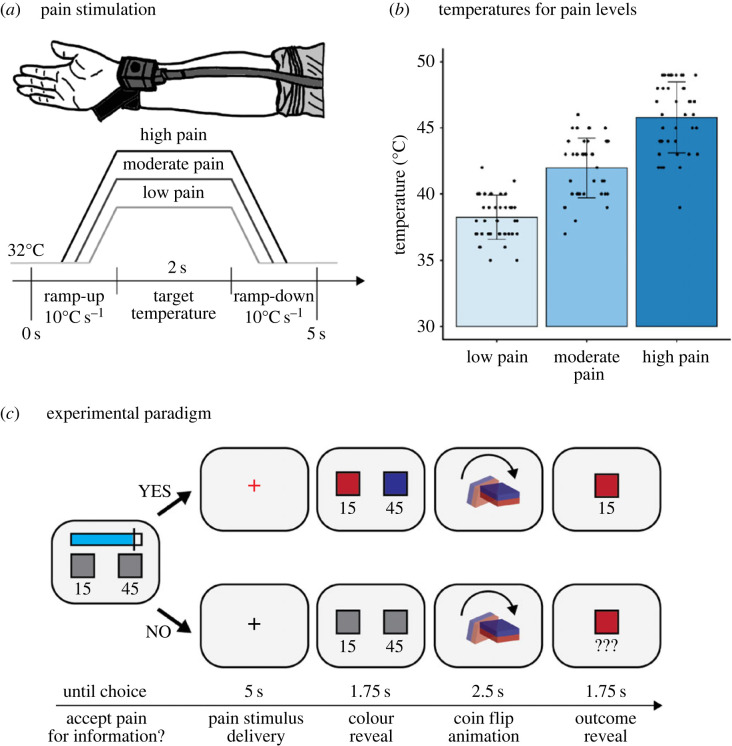
(*a*) Illustration of pain stimulation via delivery of individually calibrated low, moderately or highly painful thermal stimuli to the volar part of the forearm. (*b*) Distribution of temperatures rated as low, moderate and high pain, respectively. Single dots represent averages from individual participants. (*c*) Experimental paradigm. In each trial, participants first learned the two amounts that could be won in the coin flip lottery and the pain level to reveal the assignment of colours to amounts. If participants chose to accept the painful stimulus, the corresponding thermal intensity was delivered. We then revealed the colour assignments for the coin, which was followed by an animation of the coin flip. Finally, the outcome colour and final reward outcome were displayed. By contrast, if they rejected the painful stimulus, the colour mappings were not revealed, and the original display was represented. They observed the coin flip animations and were shown the winning colour, but without learning the final reward outcome. Regardless of their decision, however, the winnings were always added to their running total.

In this task, participants decided in each trial whether to accept low, moderate or high pain in exchange for immediate information about the winnings of a coin flip lottery. A Pathway Pain & Sensory Evaluation System (Medoc Advanced Medical Systems) was used to administer painful thermal stimuli, delivered to participants’ left forearm (figure 1*a*). Participants used the Gracely Scale [38,39] to indicate the subjective pain experience on a scale of 0 to 20. A calibration block was conducted prior to the main experiment, implementing an ascending staircase of thermal intensities, starting from a temperature of 35°C, and increasing the temperature by 1°C until participants' rating of ‘high pain’ was reached (with a set maximum of 50°C that was never reached). The calibration procedure was conducted twice. The first round served to accustom participants to the stimuli, and ratings from the second round were used to define low (5), moderate (10) and high (15) pain levels [40] (figure 1*b*). Note that high pain was always below the maximal tolerable stimulation and did not pose any danger of burning the skin. After calibration, participants were given a random sequence consisting of nine thermal stimuli, three of which corresponded to each of their pain levels, and they were asked to rate them as low, moderate or high pain. This was done to confirm that participants could correctly distinguish between pain levels before proceeding to the main task. All participants passed this check without errors.

To create the lotteries, amounts between 5 and 85 points were combined and randomly associated with either the red or the blue side of each coin, resulting in four different *expected values* (EV) (i.e. the average of both sides of the coin; 30, 40, 50, 60) and four levels of *range* (i.e. differences between reward amounts: 20, 30, 40, 50). Participants were instructed that, as with a real coin flip, the probability of each side winning was *p* = 0.5 in each trial. They were further explicitly instructed that these points would be exchanged for real money after the experiment, with a maximum of AUD10 to be won. Each of these 16 combinations was randomly selected once per experimental block.

### (c) Procedures and paradigm

First, participants were given written and illustrated instructions about the task, and we ensured that all aspects of the task were understood. Participants then provided written informed consent. Next, they underwent the calibration procedure (see above) to establish low, moderate and high pain levels.

For the main experiment, participants were seated comfortably in a chair, approximately 50 cm distance from a HP monitor (1920*1080 resolution; 60 Hz refresh rate). Their left arm rested on an arm rest, with the probe for pain stimulation secured with a velcro fastener. The experiment was presented via PsychToolbox-3 [41] running on MATLAB R2017b (The Mathworks, Natick, MA), using a HP computer (Intel Core i7-8700 processor).

In each trial, participants were shown a screen displaying both sides of the coin, together with the point values that each side would earn them if it won. However, both sides were displayed in grey, meaning that the participant did not know which colour would earn which amount. Above, participants were also shown a blue bar with a red line, indicating one of the three pain levels (low, moderate or high). Participants were instructed to choose whether they accept the pain stimulus in exchange for learning the colours associated with the point values for this coin flip, by clicking the left or right button, mapped onto the screen position ‘yes’ and ‘no’, respectively. The key press terminated the displayed screen, and a brief blank screen was shown for 1 s. If they accepted the pain stimulus, the fixation cross on the next screen turned red, and the stimulus was delivered during the next 5 s, starting at 32°C, followed by a ramp-up phase (10°C/s), 2 s target temperature stimulation, and a ramp-down phase (10°C/s). Subsequently, the associated colours and point values were displayed for 1.75 s, after which the coin flip was animated (2.5 s), and subsequently the outcome colour and the amount won were shown for 1.75 s (figure 1*c*). If the pain stimulus was not accepted, the fixation cross stayed black for the same period of 5 s (to prevent speeding up the task by rejecting the pain), but no pain stimulus was delivered. This was followed by the same display of point values, but each still paired with an uninformative grey colour. Subsequently, the coin flip was also animated, and the outcome colour was shown; however, without revealing the final amount won (figure 1*c*). Regardless of the acceptance of pain and reveal of the colour-points association, the winnings were always added to the running total, which was made clear to the participants at the start.

Participants performed six blocks of 16 trials (four expected reward values combined with four ranges; see above). Each of the three pain levels was used throughout two blocks, and the order of blocks was pseudo-randomized for each participant, with the constraint that the same pain level was not repeated across consecutive blocks. Participants were given a self-paced break of a few minutes to rest between blocks to prevent desensitization to pain, and the stimulation side was moved by a few centimetres after each block [42]. After the experiment, participants completed the Five-Dimensional Curiosity Scale-Revised (5DCR) [43] and State Trait Anxiety Inventory (STAI) [44]. Finally, they were debriefed, and reimbursed for their time.

### (d) Statistical analyses

**Figure 2.**
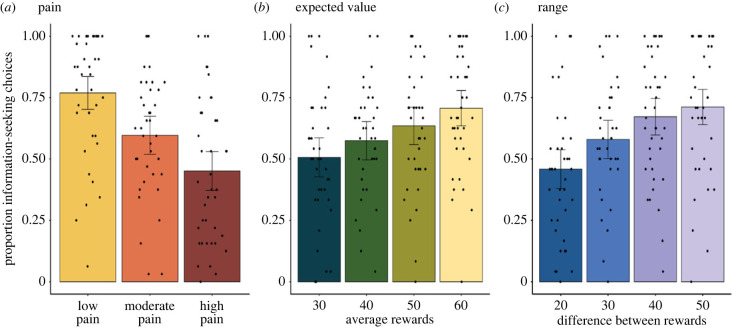
Proportion of information-seeking choices by accepting the painful stimulus by (*a*) pain level (low, moderate and high pain), (*b*) expected value of the lottery (i.e. the average reward of both sides of the coin) and (*c*) range (i.e. difference between reward values of both sides of the coin). All three main effects were significant, meaning that each of these factors predicted decisions for choosing non-instrumental information.

In figure 2*a* it can be seen that participants accepted pain in exchange for non-instrumental information for each pain level. We then performed logistic mixed model analyses using the lme4 package [45] in R (version 4.1.2) to test formally whether pain was a significant predictor of information-seeking choices. Specifically, we included pain level (i.e. price), EV and range as fixed and random (i.e. subject-level) effects (slopes), and participant ID as a random intercept. For this analysis, EV and range values were z-scored to obtain meaningful comparable beta values. We compared models with main effects only to models incorporating two-way interaction terms, and finally the full model containing all possible interaction terms, including the three-way interaction. For model convergence, all interactions were included as fixed effects.

## 3. Results

### (a) Pain stimulation

For low pain, the average temperature was 38.3°C (s.d. 1.66), with an average pain rating of 5.2 (s.d. 1.34). For moderate pain, the average temperature was 42.0°C (s.d. 2.26), with an average pain rating of 10.1 (1.50). Finally, for high pain, the average temperature was 45.8°C (s.d. 2.69), with an average pain rating of 14.9 (s.d. 1.53). Temperature differences were significant between levels, as confirmed by unsigned *t*-tests (low versus moderate pain: *t*_39_ = 23.875, *p* < 0.0001; moderate versus high pain: *t*_39_ = 23.374, *p* < 0.0001) (figure 1*b*).

### (b) Information-seeking

Participants were willing to accept pain stimuli in exchange for information for all pain levels (figure 2*a*). Control analyses showed that there were no differences in proportions of information-seeking choices between the experimental blocks featuring the same pain level, meaning that behaviour remained stable across the experiment (see electronic supplementary material, figure S1).

The mixed-effects modelling showed that the best fitting model was model 1, which included pain, EV and range, but no interactions (table 1). Pain was a significant negative predictor of choice, with the probability of accepting the painful stimulus decreasing as the intensity of the pain increased. There were also significant positive main effects of EV, with a higher average value of the two possible rewards predicting higher probability of accepting the painful stimulus (figure 2*b*), as well as range, with a higher difference between possible reward amounts predicting higher probability of information seeking (figure 2*c*) (table 2 for full results). Notably, these results show that, while all three factors independently increased information-seeking, the effect of pain on information-seeking choices did not interact with the reward on offer.

**Table 1.** Model comparison for predicting information-seeking choices. EV, expected value.

models	AIC	BIC
(1) main effects only	3041.1	3128.6
(2) main effects plus interactions for pain (EV : pain, range : pain)	3043.8	3143.9
(3) main effects plus all pairwise interactions	3045.1	3151.4
(4) main effects, all pairwise interactions, three-way-interaction	3045.3	3157.9

**Table 2.** Model 1 parameters predicting information-seeking choices. Expected value, range and pain were continuous variables. Statistics for fixed effects are reported.

model 1 (pain, EV, range as fixed effects)		
predictor	*ß* (SE)	*p*
intercept	3.99 (0.55)	<0.0001
expected value	0.65 (0.16)	<0.0001
range	0.96 (0.22)	<0.0001
pain	−1.41 (0.24)	<0.0001

### (c) Questionnaires

We correlated the individual questionnaire scores with pain sensitivity (see electronic supplementary material, tables S1 and S2 for descriptive statistics). Pain sensitivity was defined as the temperature rated as low, moderate and high pain at each pain level, respectively. In other words, individuals who indicated that a specific pain level (e.g. low pain = rating of 5) was reached at a relatively low temperature can be thought of as having a relatively high pain sensitivity (see electronic supplementary material, tables S3 and S4 for full results). Significant correlations were found for the deprivation sensitivity scale of the 5DCR, which negatively correlated with temperatures for low pain (*r* = −0.44; *p* = 0.005), moderate pain (*r* = −0.35; *p* = 0.027) and approached significance for high pain (*r* = −0.28; *p* = 0.077) (electronic supplementary material, figure S2). Note that only the correlation at low pain survived Bonferroni correction. Next, we corrected the questionnaire scores with information-seeking probability. The only notable correlations were found between proportion of information-seeking choices at low pain levels for STAI state anxiety (*r* = 0.36; *p* = 0.021) as well as trait anxiety (*r* = 0.33; *p* = 0.036). This means participants scoring high on either anxiety score chose information more often when it was available at a low pain cost (electronic supplementary material, figure S3). No significant results were found at the other pain levels for STAI scores, nor for any of the 5DCR scales (see electronic supplementary material, tables S5 and S6 for full results).

## 4. Discussion

In this study, we tested whether human participants were willing to incur pain for non-instrumental information—that is, immediate but functionally useless information—about the outcome of an insignificant, predetermined coin-flip lottery. We found that, while stronger pain was associated with less desire for information, participants nevertheless accepted even high pain in nearly half of the trials. Our mixed-effects modelling results showed that, in addition to the level of pain, the expected value, as well as the range between the two possible rewards, independently predicted the decision to seek information. There was no interaction between these factors with pain level. Importantly, for a rational agent, none of these factors should increase the desire for information. Regardless of how much was at stake, or how different the potential outcomes were, the available information could not be used to change the odds of winning. Our exploratory questionnaire results suggest that anxiety might be a predictor for information-seeking, in particular when the cost (i.e. pain level) was low.

Why do humans accept pain for information they do not need? Our results provide strong evidence that, like other animals, including non-human primates [4–7,21], humans value learning the outcome of relevant events upfront. This has been demonstrated by showing that human participants are willing to sacrifice small amounts of money [8–11] or invest physical effort [12] to obtain such information about similar lotteries to the one used here. However, investigating whether humans are also willing to incur pain to obtain such information provides a potentially more sensitive test for whether information is truly associated with intrinsic value, as pain is a biologically relevant, salient, and inherently aversive stimulus that all species actively strive to avoid [26]. Importantly, the optimal strategy in our experiment, both with respect to reward (which could not be maximized), as well as to pain avoidance, was to never accept any pain in exchange for information. Participants were clearly informed that they could not change the lottery outcomes and that not knowing the outcome had no possible negative consequences for the probability of winning. Hence, the role of the obtained information cannot be attributed to instrumental value (i.e. its potential to be used to maximize financial rewards). Our findings therefore provide clear evidence that participants are nevertheless willing to accept substantial costs to obtain such non-instrumental information.

A possible explanation for our finding is that it is not the information *per se* which is valuable, but the cognitive or affective state that is achieved when the information is obtained. The intrinsic value of obtaining the information, at least for our type of paradigm, might lie in the immediate reduction of uncertainty. In support, a series of studies, which have used variants of our lottery task in combination with computational modelling of behaviour, have found that non-instrumental information-seeking was best characterized by participants' desire for an early resolution of uncertainty [8,9], and that model fits further improved when individuals’ subjective perception of uncertainty was also modelled [12]. This suggests that it is not necessary to postulate that the non-instrumental information itself is positively valenced. Agents could simply trade off two negatively valenced internal states against each other: Either the anticipation of physical pain, or the anticipation of a prolonged state of uncertainty. Furthermore, as hypothesized, we found that a higher expected value and a greater range of rewards increased information-seeking behaviour. While it is possible that this reflects the effect of a higher possible anticipated positive outcome, which increases the subjective value of the information, it could also simply reflect that these factors increase the relevance of the state of aversive uncertainty [22].

The idea that participants might trade off the two negatively valenced internal states (i.e. the perception of pain and of uncertainty) is also plausible when considering their neural basis. Pain intensity has been found to be represented by multiple cortical and subcortical systems [28,46–50]. A mesolimbic system comprising the ventromedial prefrontal cortex (vmPFC) and nucleus accumbens (NAc) has been suggested to be involved in creating the pain experience [30,51], the dorsolateral prefrontal cortex (dlPFC) and anterior cingulate cortex (ACC) are thought to be involved in regulating the level of pain experienced [52] and the informational value of pain states [53]. The network involved in experiencing and modulating pain shows strong overlap with neural networks for processing reward, which involves a large number of cortical and subcortical regions [26,30,34,36,37,51,52]. Within some regions, such as the amygdala and ventral striatum (VS), pleasure and pain hotspots have been found to exist in close proximity [34]. In the OFC, subregions have been suggested to represent the hedonic value of both reward and punishment [34,54], as well as variables related to information-seeking ([5]; however, it should be noted that Blanchard and colleagues argue that the coding scheme in these OFC regions is not compatible with the idea of integration into a single variable). Other pain-related regions, however, have also been associated with the valuation of non-instrumental information, most notably the ventral striatum (VS), the anterior cingulate cortex (ACC) [12,23–25], and the anterior insula [33] and might therefore provide the required neural architecture for assigning intrinsic value to comparing the value of terminating one aversive state (i.e. uncertainty) by accepting another (i.e. pain). Coll *et al.* [33] recently demonstrated shared neural representations in the anterior insula for immediate pain and expected future pain, as well as for unpleasant pictures. Recent findings in *Drosophila* are also relevant as they show that punishment-sensitive dopaminergic neurons play a role in comparing which of two aversive experiences is relatively preferrable [55]. The anticipation of terminating a negatively valenced state, such as uncertainty, could also have an analgesic effect, similar to the anticipation of positively valenced experiences [34], which could further explain the high acceptance rate for pain stimuli in our study.

The idea that the desire to reduce uncertainty is valuable also provides a parsimonious explanation for the findings from the animal literature. In non-human animals, such as monkeys, starlings and pigeons [4,6,7,21], a prolonged state of uncertainty, such as whether to remain in a potentially dangerous environment with an unknown probability of obtaining water or food, might have serious consequences. Any information that helps agents to terminate this aversive state early might therefore be worth sacrificing potential rewards. Whether other animals would also accept pain, however, remains an open question.

A related explanation comes from a recent framework by Bromberg-Martin & Sharot [19], who have suggested that humans (and possibly non-human primates) have an intrinsic desire to be in a positive belief state. Receiving information about winning the lottery in a given trial would be the desired belief state, which means agents would be required to decide whether this positive belief state is more valuable than the aversive experience of pain. However, in our study, it seems unlikely that the desirable belief state (i.e. knowing whether one won a few points) was a sufficiently powerful driver for agents to repeatedly accept pain. In particular, participants *could not lose* in our lottery; they simply did not know *how much they won* in each trial. Even if one considers the smaller amount as being the ‘negative outcome’, participants were fully aware that the coin flip could only result in a ‘win’ (i.e. the larger amount) in half of all trials; hence, they could also predict that seeking information would result in an *undesirable* belief state in the other half of the trials.

After establishing the general effects, research in humans can also explore potential moderating personality factors (e.g. [20,56]). For this, future studies should employ larger sample sizes. We could not find any consistent significant correlations between information-seeking choices and the 5DCR curiosity questionnaire [43], and only significant correlations for the state and trait anxiety scales of the STAI [44] at the low pain level. Our small sample size prohibits drawing strong conclusions from these results, in particular about differences between pain levels, given that participants made most of their choices to seek information for low pain. However, the results are in line with previous research, designed to investigate personality factors in larger samples, in which people scoring high on anxiety/negative emotionality as well as high on obsessive-compulsion personality aspects were more likely to sacrifice small amounts of money to obtain non-instrumental information [9]. The personality dimension Openness/Intellect, which has been linked to curiosity for information [57], however, was not meaningfully related to non-instrumental information-seeking [58]. Interestingly, in our study, the deprivation sensitivity of the 5DCR, which measures the anxiety and frustration experienced when people cannot access information desired, correlated negatively with pain tolerance (conceptualized as the temperatures rated as low, moderate and high pain, respectively), meaning that people with low pain tolerance scored higher on this scale. Deprivation sensitivity is thought to reflect the negatively valenced aspect of curiosity, that is, the urge to terminate the unpleasant state of uncertainty, rather than joyous exploration (which corresponds to positive emotions associated with learning and growing) [43]. This finding might serve as another indicator that experiencing physical pain and experiencing uncertainty are more strongly related than previously known.

It is further reasonable to assume that the negative valence of stronger pain experiences than used in our experiment would eventually exceed the negative valence of uncertainty, but the exact threshold remains unknown. In general, pain is perceived as a noxious and highly biologically relevant experience across species. Pain has been shown to interact with cognitive processes, and directly impact decision-making and preference formation in humans and monkeys [34,36]. With thermal (e.g. [40,42]) and laser (e.g. [36]) stimulation methods becoming more practical and widely available, it is now possible to examine how the desire to avoid (or terminate) a noxious experience affects decisions and information-seeking (e.g. [33]). In particular, painful stimuli can be used to examine the role of neurotransmitters such as dopamine and serotonin to compare the value of avoiding one aversive event against another [34,55].

Another valuable avenue for future research would be the explicit inclusion of gender effects to systematically test for differences in pain acceptance. As for including any other individual differences, however, such a study would need a much larger sample to achieve sufficient statistical power. Another extension of this experiment could be to directly compare different ‘currencies’ for obtaining non-instrumental information. If some individuals value information more than others (e.g. to learn the outcome of a lottery), their willingness to pay small amounts of money should correlate with their willingness to invest physical effort or endure pain (or other currencies) in a domain-independent fashion. Alternatively, future studies could give people the option to avoid pain by paying money instead, for example, or by investing effort, to still obtain the information, which would allow for deriving psychometric functions that can map these currencies onto the same space. Another unresolved question is whether experimental scenarios can be constructed in which participants might want to avoid learning the outcome of a lottery, because it is always negative, and whether they are willing to endure pain in return. This would be in line with studies demonstrating that people often prefer learning good news but might want to remain ignorant about bad news, in particular if the bad news are potentially threatening [14,17,59–61].

Future research should also explore the neural correlates of decisions to seek non-instrumental information in exchange for pain. The next step could be an investigation into whether neural representations of anticipated pain and the subjective value of reducing uncertainty can be found in the same neural systems, as suggested by previous research (e.g. [26,33,37]). In particular the ACC, which has been separately linked to the processing of conflict, reward, pain regulation and non-instrumental information-seeking in humans and monkeys [12,23–25,30,52], is a key candidate for this trading-off the subjective value of enduring pain and being able to reduce uncertainty. However, recent work also points to a potential role of the anterior insula for representing different unpleasant experiences [33].

## 5. Conclusion

In summary, we found that humans were willing to accept various levels of physical pain in exchange for immediate information about small winnings in a series of predetermined lotteries. This behaviour can be explained by assuming that the uncertainty that comes from not knowing is intrinsically aversive, and it is possible for participants to trade off the value of terminating this state against another, short-lived aversive state, which is physical pain. This raises the intriguing question of how the brain transforms valence-related signals, reflecting both aversive and rewarding factors, into a common code to trade them off against each other at every moment in time.

## Data Availability

Data and code are freely available on OSF https://osf.io/nvpha/. Additional information is provided in electronic supplementary material [62].
